# Supplementary material for: Coherent transfer of electron spin correlations assisted by dephasing noise
Source: Nat Commun. 2018 May 30;9:2133. doi: 10.1038/s41467-018-04544-7 (PMC5976655; doi:10.1038/s41467-018-04544-7)
Supplement: Supplementary file 1 — Supplementary Information [file 41467_2018_4544_MOESM1_ESM.pdf]

Coherent transfer of electron spin correlations assisted by dephasing noise

Takashi Nakajima *et al.*

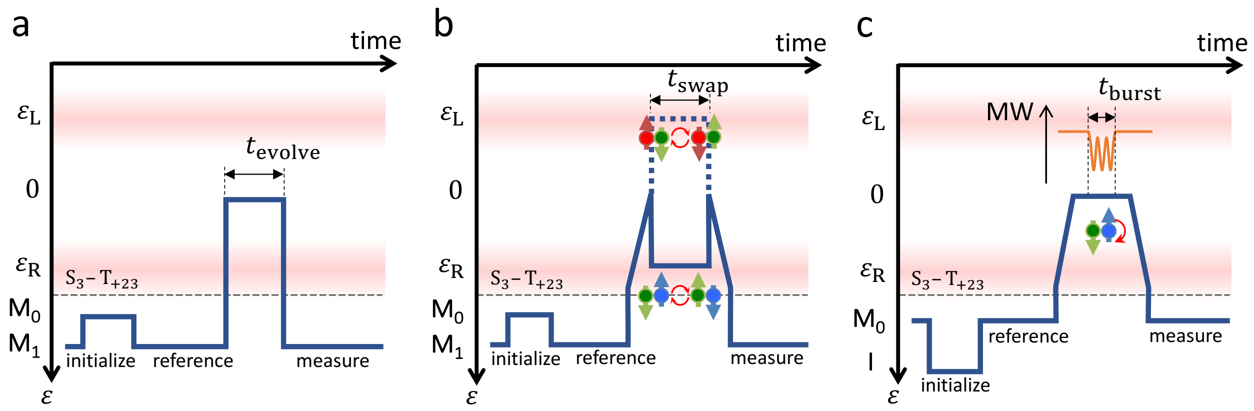

Supplementary Figure 1 Pulse sequences used in the experiment. a, Pulse sequence used in the measurement of the coherent evolution of the entangled states in Figs. 3 and 4. **b,** Sequence for the dephasing measurement in Fig. 5. **c,** Sequence for the ESR measurement in Supplementary Fig. 2.

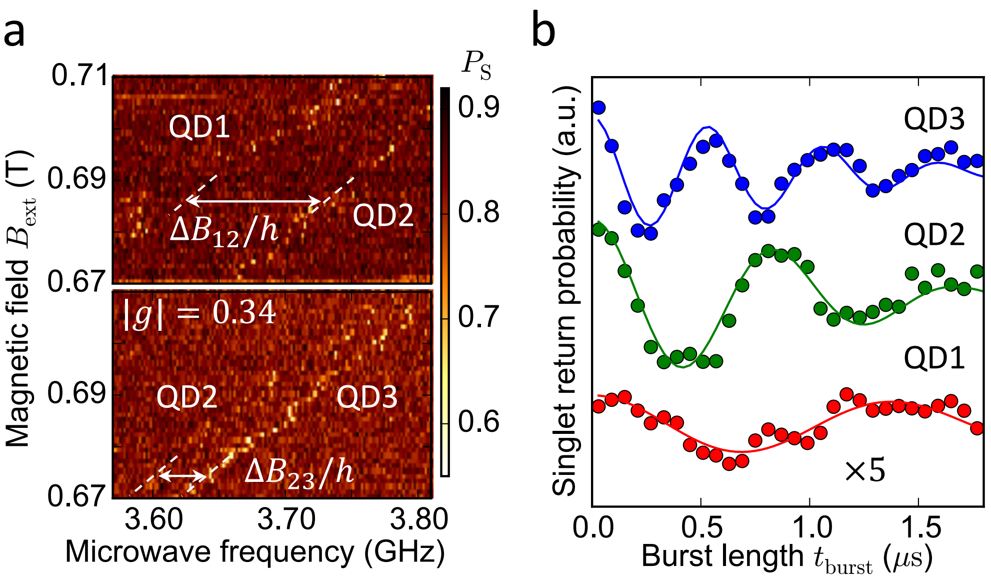

Supplementary Figure 2 ESR and Rabi oscillations of the spin qubits. a, ESR signals measured as a function of B_{ext} and the microwave frequency for QD1 and QD2 (upper panel) and QD2 and QD3 (lower panel). Note that the resonance frequency of QD2 in the upper panel is shifted from the one in the lower panel because the gate bias conditions (and consequently the local Zeeman field) are slightly changed to improve the visibility of the spin blockade signal near the (111)-(201) charge transition. **b**, Rabi oscillations of QD1 (magnified fivefold), QD2 and QD3 driven by the application of a microwave burst of length t_{burst} at $B_{\text{ext}} = 0.7\,\text{T}$. The singlet return probability P_{S} is derived from 60 consecutive sweeps of t_{burst} and then smoothed by taking a sliding Gaussian average.

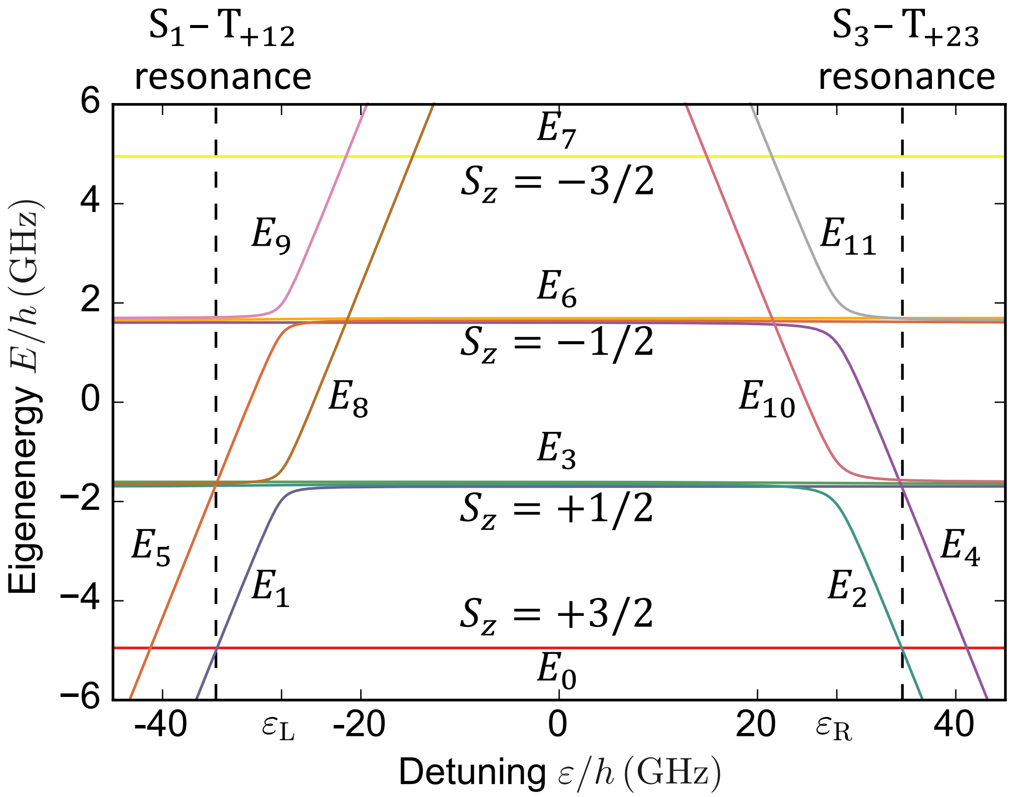

Supplementary Figure 3 Energy diagram of the three-spin states. All of the eight three-spin energy eigenstates calculated from Eq. (2) are shown. Expanded views of the $S_z = \pm 1/2$ branches are shown in Fig. 2d. The detuning values of the singlet-triplet resonance points near the (201)-(111) and (111)-(102) charge transitions are shown with dashed lines.

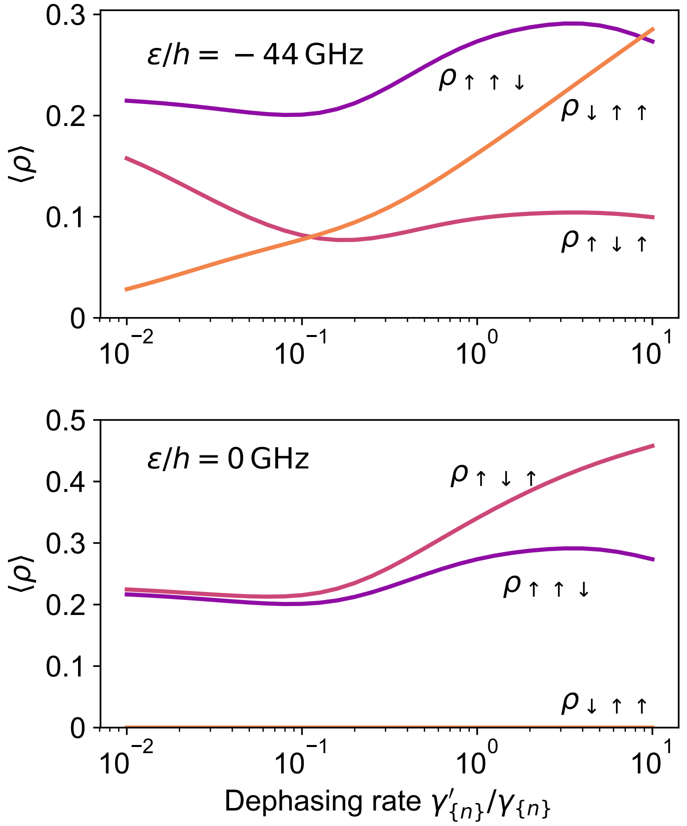

Supplementary Figure 4 Population of the spin states in the dephased transition process. Density matrix elements $\rho_{\sigma_1\sigma_2\sigma_3} = \langle\sigma_1\sigma_2\sigma_3|\,\rho\,|\sigma_1\sigma_2\sigma_3\rangle$ after ($\varepsilon/h = -44\,$GHz, upper panel) and during ($\varepsilon = 0$, lower panel) the transition process with the detuning pulse toward $\varepsilon/h = -44\,$GHz are plotted as a function of the dephasing rates $\gamma'_{\{n\}}$ ($\{n\} = \{\varepsilon_\mathsf{L}, \varepsilon_\mathsf{R}, t_\mathsf{L}, t_\mathsf{R}\}$). The dephasing rates are varied simultaneously from $10^{-2}\gamma_{\{n\}}$ to $10\gamma_{\{n\}}$, where $\gamma_{\{n\}}$ are the values found in the main text.
